# Preterm birth alters the gut microbiota, metabolome and health outcomes of twins at 12 months of age

**DOI:** 10.3389/fcimb.2025.1700965

**Published:** 2026-01-21

**Authors:** Hong Mei, Liqin Hu, Meng Yang, Feiyan Xiang, Hao Zheng, Xiaonan Cai, Guilin Hou, Ruizhen Li, An’na Peng, Jianduan Zhang, Ai’fen Zhou, Han Xiao

**Affiliations:** 1 Institute of Maternal and Child Health, Wuhan Children’s Hospital (Wuhan Maternal and Child Healthcare Hospital), Tongji Medical College, Huazhong University of Science and Technology, Wuhan, Hubei, China; 2 Department of Child Healthcare, Wuhan Children’s Hospital (Wuhan Maternal and Child Healthcare Hospital), Tongji Medical College, Huazhong University of Science and Technology, Wuhan, Hubei, China; 3 Child Healthcare Department for Community, Wuhan Children’s Hospital (Wuhan Maternal and Child Healthcare Hospital), Tongji Medical College, Huazhong University of Science and Technology, Wuhan, Hubei, China; 4 Department of Maternal and Child Health Care, School of Public Health, Tongji Medical College, Huazhong University of Science and Technology, Wuhan, Hubei, China; 5 Department of Obstetrics, Wuhan Children’s Hospital (Wuhan Maternal and Child Healthcare Hospital), Tongji Medical College, Huazhong University of Science and Technology, Wuhan, Hubei, China

**Keywords:** gut microbiota, infant health, metabolome, preterm birth, twin

## Abstract

**Introduction:**

Perinatal factors affect gut microbiota and infant health, but the combined impacts of preterm birth and chorionicity on gut microbiota, metabolism, and physical/neurobehavioral development in twins remain unclear.

**Methods:**

A total of 143 twin families (12-month-old infants) were enrolled and divided into four groups by gestational age and chorionicity (dichorionic-diamniotic full-term/preterm, monochorionic-diamniotic full-term/preterm). Gut microbiota diversity and fecal metabolism were analyzed via 16S rRNA sequencing and untargeted metabolomics. Wilcoxon’s rank-sum tests, generalized estimating equations, and twin-based ACE models were used for alpha diversity comparison, differential microbiota identification, and genetic/environmental contribution evaluation, respectively, with confounder adjustment in development association analysis.

**Results:**

We identified 10 group-specific gut microbiota genera and 394 differential metabolites. Fifty-two microbiota taxa showed genetic variance, but none overlapped with group-specified taxa. Six genera and 18 metabolites correlated with twins’ physical/neurobehavioral development. Pathway analysis highlighted three key metabolites: Morphine (isoquinoline alkaloid biosynthesis, drug metabolism, neuroactive ligand-receptor interaction), Nicotinuric acid (nicotinate/nicotinamide metabolism), and Catechin (flavonoid/phenylpropanoid biosynthesis).

**Discussion:**

Preterm birth is linked to gut microbiota dysbiosis and metabolic perturbations, which may affect twin development. Notably, preterm birth exerts a stronger effect than genetic factors in shaping the gut microbiota of 12-month-old twins.

## Introduction

Preterm birth (PT) generally refers to birth with a gestational age below 37 weeks. PT affects approximately 10% of babies worldwide (Huang et al., 2023), and the PT rate in twin pregnancies can reach 35% or higher (Geisler et al., 2014). PT has short- and long-term health consequences on the human body. For example, surviving preterm babies are at greater risk for central nervous system, hearing, and vision problems at birth (Romero et al., 2018). Furthermore, premature birth is an independent predictor of short- and long-term respiratory and cardiovascular risks (Starr et al., 2022; Burchert et al., 2023). For children at an early age, PT has a widely spanned impact on anthropometric growth, cognitive development, and behavior (Gomaa et al., 2022; Talej et al., 2022; Bando et al., 2023).

As a research topic of utmost interest in recent years, the human microbiota has been documented to play non-negligible roles in the gut-systemic metabolic interplay, which may influence immune and metabolic development in early life and alter offspring cognitive development and behavior through the microbiota-gut-brain axis (Stiemsma and Michels, 2018; Visconti et al., 2019; Fan and Pedersen, 2021). Many studies indicate that host genetics and PT can shape the microbiome at birth and persistently influence the pace of microbial acquisition before getting an adult-like gut microbiome construction (Tamburini et al., 2016; Hill et al., 2017; Fouhy et al., 2019). Twins have high genetic and environmental resemblance, as monozygotic twins share 100% of their genes compared to dizygotic twins with 50% shared genes, and they are characterized by the common environment of parents and family, like parenting style, education, and so on (Park et al., 2022). These similarities make twins an excellent model for disentangling the effect of genetic and environmental factors on shaping gut microbiota. However, most twin studies on the genetic and environmental determinants of the gut microbiota have focused on genetic effects (Goodrich et al., 2014; Goodrich et al., 2016); twin studies on the association between genetic and environmental factors and the gut microbiota are rare (Turnbaugh et al., 2009; Yang et al., 2020).

Apart from the zygosity characteristics, twins have chorionicity, which can be classified as dichorionic-diamniotic and monochorionic-diamniotic twins. Approximately 20% of twins are monochorionic-diamniotic, and most of them are monozygotic. Several studies have found that adverse perinatal outcomes among twins differ significantly depending on chorionicity (Adegbite et al., 2005; Lewi et al., 2008; D’Antonio et al., 2017), among which monochorionic-diamniotic imparts a greater risk of PT than dichorionic-diamniotic twins (Marleen et al., 2021). PT in dichorionic-diamniotic twins could be attributed to various genetic or/and environmental factors, including genetic dissimilarity and differences in placental mass, placental insufficiency, umbilical venous diameter, and flow (Couck et al., 2022; Eaves et al., 2023; Roberts et al., 2023). On the contrary, monochorionic-diamniotic twins share more genetic resemblance, as most of them are monozygotic pairs, and the particular challenges of PT in monochorionic-diamniotic twins arise mainly from the shared placenta and the placental vascular anastomoses, which are almost universal (National Institute for Health and Care Excellence: Guidelines, 2019). The correlation between chorionicity and PT makes twin pairs ideal for investigating placental-related long-term growth and development in children and how genetic and environmental factors shape the gut microbiome and metabolism. Therefore, well-designed twin studies are required to elucidate the impact of genetic and intrauterine environmental factors on gut microbiota.

Based on the Wuhan Twin Birth Cohort study, we collected fecal samples from twin pairs at 12 months of age and used them for 16S rRNA gene sequencing in 268 twins and for untargeted metabolomics in 138 twins, and we measured twins’ anthropometrics and neurobehavioral development at 12 months of age. Then we classified the twins into four groups based on their gestational age at birth and chorionicity: the dichorionic-diamniotic full-term birth (DCFT) group, the dichorionic-diamniotic PT (DCPT) group, the monochorionic-diamniotic full-term birth (MCFT) group, and monochorionic-diamniotic PT (MCPT). The first goal of this study was to determine the effect of gestational age at birth and chorionicity on gut microbiota and metabolism of twins at 12 months of age. Then, to evaluate the effect of microbiota and metabolites on physical and neurobehavioral development at 12 months of age.

## Materials and methods

### Ethics approval and consent to participate

This study was approved by the Ethics Committee of Wuhan Children’s Hospital (Wuhan Maternal and Child Healthcare Hospital) (IRB number: WHFE2016050) and performed according to relevant guidelines and regulations. Informed consent from the infants’ parents was obtained before the investigation.

### Subject recruitment

This study was conducted based on the ongoing Wuhan Twin Birth Cohort study. Mothers were approached for informed consent between March 2016 and December 2020 at Wuhan Children’s Hospital in Wuhan, China. The inclusion criteria for this sub-study contained (1) live neonates from dichorionic-diamniotic and monochorionic-diamniotic twin pregnancies, (2) twin pairs followed up at 12 months of age, and (3) twin pairs with fecal samples and anthropometric measurements or neurobehavioral estimates included. The exclusion criteria included (1) mothers with gestational diabetes mellitus or gestational hypertension during pregnancy; (2) fetuses with twin-to-twin transfusion syndrome; (3) neonates with birth defect; and (4) only one of the twins had complete data on fecal samples, anthropometric measurements, or neurobehavioral estimates.

### Data collection

Twins’ gestational weeks at delivery and chorionicity of placenta were collected from the birth record in the electronic medical record system. Gestational age at delivery was calculated as the difference between the twins’ birth date and the maternal last menstrual date. Twins can be classified as PT for gestational age <37 gestational weeks, FT for gestational age ≥37 gestational weeks, dichorionic-diamniotic, and monochorionic-diamniotic twins. We further calculated the twins into DCPT, DCFT, MCPT, and MCFT groups according to their gestational age at birth and chorionicity. Twins’ sex, weight, length, and neurobehavior were measured at 12 months of age. Twins’ neonatal blood spot samples were collected. According to the short tandem repeats genetic typing technique on blood spot samples, twins’ zygosity was detected as monozygosity (MZ) or dizygosity (DZ). Twins’ sex was set as boy or girl. Body mass index (BMI) was calculated as weight (kg) divided by the square of the height (m^2^). Sex- and age-specified weight, length, and BMI were generated as the weight-for-age z-score (WFAZ), length-for-age z-score (LFAZ), and BMI z-score (BMI_Z) according to the WHO Child Growth Standards (2006) (WHO, 2006). Twins’ neurobehavioural developments at 12 months of age were evaluated using the Age and Stage Questionnaire, 3rd version (ASQ-3) (Wei et al., 2015). ASQ-3 contained five development domains, including communication, gross motor, fine motor, problem-solving, and personal social, with 60 points possible in the combined evaluation for each infant. For each domain of the ASQ-3, infants’ neurobehavioral development was set as normal development, suspected development delay, or development delay. Any domain screened > −1 SD was defined as normal development (ND), screened between −2 SD and −1 SD was defined as suspected development delay, and < −2 SD was defined as developmental delay. As the proportion of development delay domains in our participants was small, we merged suspected development delay and development delay as development delay (DD). To fully assess the neurocognitive development, we generated a variable as a neurocognitive problem, set as yes or no. The answer “yes” was defined as at least one of the five development domains being DD, and the answer “no” was defined as all five development domains being ND. Apart from these leading indicators, we also collected parental BMI, maternal educational level, maternal age at delivery, delivery mode, whether assisted reproductive technology (ART) was used, twins’ birth weight, feeding patterns at the 1st and 6th months, and antibiotics used during the first 6 and 12 months of life.

### Fecal sampling, DNA extraction, and high-throughput sequencing

Fresh fecal samples were collected in a sterile plastic container by caregivers 1 or 2 days before the interview and immediately frozen at −20°C at home before being transferred to the −80°C freezer within 1h of arriving at the study hospital. DNA extraction was conducted using the QIAamp PowerFecal DNA Kit (QIAGEN, Germantown, MD, United States), and high-throughput sequencing was provided by Novogene Co. Ltd. (Beijing, China). DNA extraction and 16S rRNA gene high-throughput sequencing in the V3–V4 region details were illustrated in one of our previous studies (Mei et al., 2022).

### Microbial community analysis

We used QIIME (version 1.9.1, http://qiime.org/scripts/split_libraries_fastq.html) to assess group-specified alpha diversity, including the Shannon and Chao1 index. We assessed sample species complexity via Bray–Curtis distance and phylogenetic differences (sensitivity analysis) via weighted/unweighted UniFrac distance. Permutational multivariate analysis of variance (PERMANOVA) and permutational dispersion test (PERMDISP) were run using the vegan (2.6-10) package in R 4.3.3. The Wilcoxon rank sum test identified significant differences in Shannon/Chao1 indexes and species complexity across DCPT, DCFT, MCPT, and MCFT groups. Principal coordinate analysis (PCoA) distinguished group-specific signatures (primary: Bray–Curtis distance; sensitivity: weighted/unweighted Unifrac distance), with PERMANOVA and dispersion tests applied to each metric. Alpha and beta diversity were further analyzed in subgroups stratified by gestational age, chorionicity, and zygosity. For taxonomic diversity analysis: (1) only genera taxa presented in >10% of fecal samples at 12 months old were retained; (2) genus-level relative abundances were log_2_ transformed (after adding 1 to zero values) to achieve normality (verified via normality test). Differentially abundant genera across the four groups (adjusted for confounders) were identified using generalized estimating equations (GEE) with twin pairs as random effects. All the above analyses were conducted in R 4.3.3. Means of log-transformed relative abundance for differential genus taxa in the four groups were clustered using GraphPad Prism 8.0.1.

### Heritability of the microbiome

The twin-based additive genetic, common environmental, and unique environmental model (ACE model) was used to evaluate the contribution of genetic and environmental effects on the composition and function of the gut microbiota according to methods from Jing Yang et al (Yang et al., 2022). The ACE model partitions the total variance of gut microbiota into three components: A for additive genetic effect, C for common/shared environment effect, and E for unique environment effect by comparing trait variability in MZ twin pairs versus DZ twin pairs. Before calculating the microbial heritability, raw count tables were filtered and transformed as follows. First, data in the ACE models were collected from paired MZ and DZ twins. Furthermore, twins’ sex, birth weight, and delivery mode were adjusted for in the models. Then, heritability estimations of all the 188 microbiota at the genus level were calculated. Meanwhile, the distribution of ACE model types for valid taxa and the ACE effect contribution of the top 30 taxa were also calculated. All the analyses were performed in R 4.3.3 with the OpenMx package.

### Untargeted metabolomics analysis of fecal samples

Metabolite analysis of fecal samples of 12-month-old twins, including metabolite extraction and data preprocessing was performed by Novogene Co., Ltd. (Beijing, China). Detailed methods were attached in the supplementary. Metabolites were log2 transformed to achieve normal distribution after the normality test. To detect functions of metabolites, the Kyoto Encyclopedia of Genes and Genomes (KEGG) database (https://www.kegg.jp/kegg/kegg2.html) was used. The metabolic pathways enrichment of metabolites was performed as follows: when ratios were satisfied by x/n > y/N, metabolic pathways were considered as enrichment; when the false discovery rate adjusted *p*-value of the metabolic pathway was <0.05, the metabolic pathway was considered as statistically significant enrichment.

### Statistical analysis

R 4.3.3 was used for all the analysis below. Intergroup comparisons, including microbial alpha and beta diversity, relative abundance, and culmulative log-transformed relative abundance; between DCPT, DCFT, MCPT, and MCFT groups, between dichorionic-diamniotic and monochorionic-diamniotic groups; and between full-term birth (FT) and PT groups, were conducted with Wilcoxon’s rank-sum test with multiple testing correction. To explore the effect of PT and chorionicity on infant growth, analysis of variance (ANOVA) and chi-square tests were used to explore the differences between WFAZ, LFAZ, WFLZ, and BMI_Z and the differences of the five neurocognitive development domains (communication, gross motor, fine motor, problem-solving, and personal social) among the four groups.

To adjust for the twin pair effect, GEE models with twin pairs as random effects were introduced for association analyses in our study. To control the impact of confounding factors on the study results, we first screened control variables via literature review and stepwise regression. Then, combined with directed acyclic graphs (**Supplementary Figure S1**), we finally determined the adjustment indicators for each model as follows: twins’ sex, birth weight and delivery model were filtered as confounders for group-physical growth associations, WFAZ, LFAZ, WFLZ and BMI_Z; twins’ sex, birth weight, delivery mode, and maternal educational level were confounders for group-neurobehavioural developments associations; twins’ sex, birth weight, delivery model, and antibiotic used during the first year of age were confounders for group-specific taxa, group-difference metabolites associations, specific taxa-physical growth/neurobehavioral developments associations, specific metabolites-physical growth/neurobehavioral developments associations. Bonferoni correction was used for *p-*values in multiple testing. Partial correlation analyses (controlling for twin pair, sex, birth weight, and delivery mode) assessed relationships between (1) group-specific bacterial genera and differential metabolites, (2) these genera and physical/neurodevelopmental indicators, and (3) differential metabolites and these indicators. Analyses and visualizations were performed in R 4.3.3 using the ggm and corrplot packages.

## Results

We included 119 twin pregnancy cases and tracked them for 12 months, collecting data on their gut microbiome, metabolome, anthropometrics, and neurobehavior. Among the 119 twin pairs, 67 pairs also had fecal metabolome data. Details of the main variables collected were included in **Table 1**. As shown in **Table 2**, consistent findings were observed across both the full sample cohort and the subset with available fecal metabolome data: maternal pre-pregnancy body mass index (BMI) was significantly higher in the DCPT and MCPT groups compared to the DCFT and MCFT groups (*p* < 0.05). Maternal educational level and delivery age were also significantly different among the four groups, with ART significantly higher in the dichorionic-diamniotic groups than in the monochorionic-diamniotic groups (*p* < 0.05). However, no significant difference was found in paternal BMI, delivery mode, child sex, feeding pattern at 1st and 6th months, and antibiotics used at 6th and 12th months (*p* > 0.05).

**Table 1:** Sample information at 12 months old (*n* = 143).

Variable	DCFT group (n)	DCPT Group (n)	MCFT Group (n)	MCPT Group (n)	Twin pairs (n)	Total sample size (n)
Gut microbiome	124	52	40	22	119	238
Gut metabolome	74	30	16	14	67	134
Anthropometric measurements	124	52	40	22	119	238
Neurobehavior	114	46	34	16	105	210

*n* refers to number of twins, DCFT refers to dichorionic-diamniotic full-term twins, DCPT refers to dichorionic-diamniotic preterm term twins, MCFT refers to monochorionic-diamniotic full-term twins, and MCPT refers to monochorionic-diamniotic preterm term twins; preterm term was defined as delivery with a gestational age less than 37 weeks.

**Table 2:** Basic characteristics of participants in the four groups.

Variable	Total sample size (*n* = 238)					Participants with metabolism data (*n* = 134)				
	DCFT	DCPT	MCFT	MCPT	*P*-value	DCFT	DCPT	MCFT	MCPT	*P*-value
Maternal pre-pregnant BMI (kg/m^2^); mean (SD)	21.27 (2.73)	21.84 (2.87)	21.44 (2.71)	21.87 (3.73)	0.632	21.11 (2.25)	21.96 (2.95)	21.21 (1.60)	21.97 (4.20)	0.457
Paternal BMI (kg/m^2^); mean (SD)	23.89 (3.95)	24. 10 (4.79)	25.32 (3.34)	22.69 (3.37)	0.084	23.69 (3.00)	24.84 (5.33)	24.92 (2.63)	21.77 (2.22)	0.09
Maternal educational level; *n* (%)					0.013					0.022
Middle school or less	62 (50.00)	18 (34.62)	10 (25.00)	10 (45.45)		42 (56.76)	16 (53.33)	2 (12.50)	6 (42.86)	
High school	50 (40.32)	20 (22.29)	24 (60.00)	8 (36.36)		26 (35.14)	8 (26.67)	10 (62.50)	6 (42.86)	
College or above	12 (9.68)	14 (26.92)	6 (15.00)	4 (18.18)		6 (8.11)	6 (20.00)	4 (25.00)	2 (14.29)	
Maternal age at delivery (year); mean (SD)	31.41 (3.15)	31.41 (3.45)	29.56 (4.98)	29.02 (3.10)	0.002	32.03 (3.23)	31.67 (2.32)	31.01 (6.44)	28.74 (3.55)	0.021
ART; *n* (%)	60 (48.39)	22 (42.31)	2 (5.00)	2 (9.09)	<.001	36 (48.65)	12 (40.00)	16 (100)	11 (78.57)	<.001
C-section delivery rate; *n* (%)	122 (98.39)	46 (88.46)	40 (100.00)	20 (90.91)	0.008	72 (97.30)	28 (93.33)	16 (100)	12 (85.71)	0.218
Zygosity; *n* (%)					<.001					<.001
Mono-zygosity	12 (9.68)	4 (7.69)	38 (95.00)	22 (100.00)		6 (8.11)	2 (6.67)	14 (87.50)	14 (100.00)	
Di-zygosity	112 (90.32)	48 (92.31)	2 (5.00)	0 (0.00)		68 (91.89)	28 (93.33)	2 (12.50)	0 (0.00)	
Child sex; n (%)					0.921					0.470
Boy	63 (50.81)	29 (55.77)	20 (50.00)	12 (54.55)		39 (52.70)	14 (46.67)	8 (50.00)	10 (71.43)	
Girl	61 (49.19)	23 (44.23)	20 (50.00)	10 (45.45)		35 (47.30)	16 (53.33)	8 (50.00)	4 (28.57)	
Gestational age (week); mean (SD)	37.49 (0.39)	35.30 (1.66)	37.46 (0.46)	35.56 (1.34)	<.001	37.51 (0.39)	35.89 (0.86)	37.46 (0.55)	35.31 (1.58)	<.001
Birth weight (g); mean (SD)	2646.13 (322.45)	2316.35 (449.14)	2625.50 (351.50)	2253.64 (402.20)	<.001	2673.92 (316.73)	2488.67 (420.80)	2598.75 (351.30)	2255.00 (462.38)	<.001
Feeding pattern at 1^st^ month old; *n* (%)					0.980					0.302
Exclusive breastfeeding	10 (8.06)	3 (5.77)	4 (10.00)	2 (9.09)		8 (10.81)	3 (10.00)	1 (6.25)	0 (0.00)	
Mixed feeding	102 (82.26)	42 (80.77)	32 (80.00)	18 (81.82)		60 (81.08)	23 (76.67)	15 (93.75)	12 (85.71)	
Formula feeding	12 (9.68)	7 (13.46)	4 (10.00)	2 (9.09)		6 (8.11)	4 (13.33)	0 (0.00)	2 (14.29)	
Feeding pattern at 6^th^ months old; *n* (%)					0.297					0.073
Exclusive breastfeeding	2 (1.75)	2 (4.00)	1 (2.78)	0 (0.00)		0 (0.00)	2 (7.14)	0 (0.00)	0 (0.00)	
Mixed feeding	61 (53.51)	22 (44.00)	25 (69.44)	11 (55.00)		37 (56.06)	14 (50.00)	13 (81.25)	6 (42.86)	
Formula feeding	51 (44.74)	26 (52.00)	10 (27.78)	9 (45.00)		29 (43.94)	12 (42.86)	3 (18.75)	8 (57.14)	
Antibiotic used at 6^th^ months old; *n* (%)	38 (30.65)	16 (30.77)	10 (25.00)	8 (36.36)	0.141	23 (31.08)	11 (36.67)	4 (25.00)	7 (50.00)	0.477
Antibiotic used at 12^th^ months old; *n* (%)	42 (33.87)	20 (38.46)	10 (25.00)	6 (27.27.00)	0.330	23 (31.08)	11 (36.67)	4 (25.00)	7 (50.00)	0.477

DCFT refers to dichorionic-diamniotic full-term twins; DCPT refers to dichorionic-diamniotic preterm term twins; MCFT refers to monochorionic-diamniotic full-term twins; and MCPT refers to monochorionic-diamniotic preterm term twins; preterm term was defined as delivery with a gestational age less than 37 weeks; BMI: body mass index; ART: assisted reproductive technology.

### PT outweighs genetics in shaping twins’ gut microbiota at 12 months old

**Figure 1:**
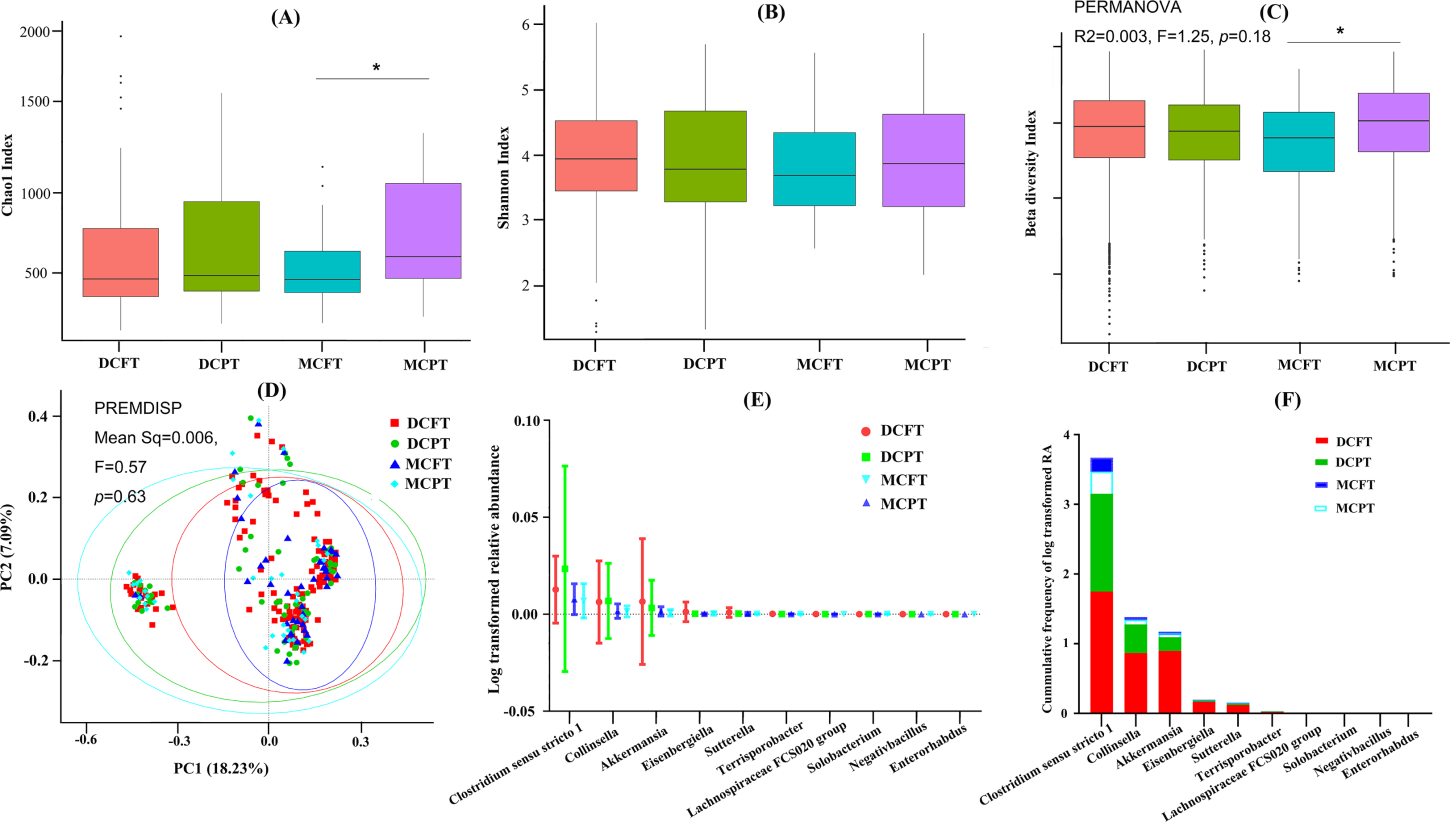
Influence of gestational age and chorionicity on the colonization of gut microbiota. DCFT refers to dichorionic-diamniotic full-term twins, DCPT refers to dichorionic-diamniotic preterm term twins, MCFT refers to monochorionic-diamniotic full-term twins, and MCPT refers to monochorionic-diamniotic preterm term twins. **(A)** The comparison of Chao1 index of gut microbiota among DCFT, DCPT, MCFT, and MCPT groups. *refers to significant difference between groups, *p* < 0.05. **(B)** The comparison of Shannon index of gut microbiota among the four groups. **(C)** Bray–Curtis distances were calculated and compared among the four groups. *refers to significant difference between groups, *p* < 0.05. **(D)** Principal component analysis plot of gut microbiota for twins in the four groups. Ellipses represent a 95% CI. **(E)** The average of log-transformed relative abundance for the 10 specified gut microbiota of the four groups. **(F)** The cumulative frequency of log-transformed relative abundance for the 10 specified gut microbiota of the four groups. RA refers to relative abundance.

Based on the group rule, we first examined whether gestational age at birth and chorionicity could affect microbial communities in twins at 12 months of age. We observed that the Chao1 index of alpha diversity of the gut microbiota in the MCPT group was significantly higher than that in the MCFT group (*p* = 0.03) (**Figure 1A**). However, no significant difference was found in the Shannon index among the four groups (**Figure 1B**). Subgroup analysis for PT and FT groups showed a significantly higher Chao1 index for the PT group (**Supplementary Figure S2A**), while no significant difference was found in the Shannon index between the two groups (**Supplementary Figure S2B**). Subgroup analysis in chorionicity found no significant difference in Chao1 and Shannon indexes (**Supplementary Figures S2C, D**). These results suggested that PT outweighs genetics in shaping gut microbiota richness in 12-month-old twins.

Next, we calculated the Bray–Curtis dissimilarity of the microbial community between individuals of each group (**Figure 1C**). The beta diversity index (Bray–Curtis distance) in the MCPT group was significantly higher than that in the MCFT group (*p* < 0.001). For dichorionic-diamniotic twins, the Bray–Curtis distances were higher in the DCFT twins than in the DCPT twins (*p* = 0.002). Consistent trends were also observed: DCFT exhibited higher beta diversity than MCFT (*p* < 0.001), while DCPT showed lower beta diversity than MCPT (*p* < 0.001). Sensitivity analyses of beta diversity with weighted UniFrac distances showed similar results, while results from unweighted UniFrac distances indicated higher distance in DCPT than DCFT groups (**Supplementary Figures S3A, B**). Finally, subgroup analysis was conducted to confirm the difference according to gestational age at birth, chorionicity, and zygosity. The Bray–Curtis distances between samples in the PT group were significantly higher than those in the FT group (*p* < 0.001) (**Supplementary Figure S3C**). In addition, the Bray–Curtis distances between samples in the dichorionic-diamniotic group were higher than those in the monochorionic-diamniotic group (*p* < 0.001) (**Supplementary Figure S3D**). In addition, a significant difference was found between monozygotic and dizygotic twins in weighted distance (**Supplementary Figure S3E**), while no difference was found in unweighted distance (**Supplementary Figure S3F**). These results indicated that PT and dichorionic-diamniotic twins might have higher heterogeneity between individuals. Additionally, the PERMANOVA analysis for all the comparison groups (**Figure 1C**, **Supplementary Figures S3A–F**) yielded small *R*^2^ values and insignificant *p*-values (*p* > 0.05), which indicated that the group variable explained only a negligible proportion of the microbial community dissimilarity (*p* > 0.05).

PCoA was used to further investigate the clustering effect of microbial communities among the four groups, DCPT, DCFT, MCPT, and MCFT. Samples were similar within each of the four groups and separated between different groups using Bray–Curtis distance (*p* < 0.001), with the distribution of DCFT and DCPT samples relatively more dispersed than that of MCFT and MCPT samples (**Figure 1D**). Similar results were shown for PCoA in weighted and unweighted UniFrac distance groups (**Supplementary Figures S4A, B**). Subgroup analysis found that the bacterial signatures in PT twins were significantly distinct from that in the FT twins (*p* < 0.001) (**Supplementary Figure S4C**), while no significant difference was found between dichorionic-diamniotic and monochorionic-diamniotic groups (*p* = 0.20) (**Supplementary Figure S4D**). Significant difference of zygosity subgroups in unweighted distance groups (*p* < 0.001) (**Supplementary Figure S4F**), while no difference was found in weighted distance groups (*p* = 0.32) (**Supplementary Figure S4E**). These results indicated that PT might lead to the distinctions among individuals outweighing genetic effects.

The GEE model identified 10 genera in four groups, DCFT, DCPT, MCFT, and MCPT, with different relative abundances (*p* < 0.05): *Akkermansia*, *Eisenbergiella*, *Sutterella*, *Lachnospiraceae FCS020 group*, *Terrisporobacter*, *Negativibacillus*, *Clostridium sensu stricto1*, *Collinsella*, *Solobacterium*, and *Enterorhabdus*. Log-transformed means and standard deviations were shown in **Figure 1E**. The cumulative frequency of the 10 discriminatory genera in the four groups was displayed in **Figure 1F**. **Figure 1F** shows that the genera *Akkermansia*, *Eisenbergiella*, and *Sutterella* were among the microbiota with the highest cumulative frequency of log-transformed RA, and the cumulative frequency of the DCFT groups was significantly larger than that in the MCFT groups (*p* < 0.05). These results further confirmed that chorionicity significantly shapes the gut microbiota in 12-month-old twins.

### Specific taxa correspond to the environment or genetic factors

**Figure 2:**
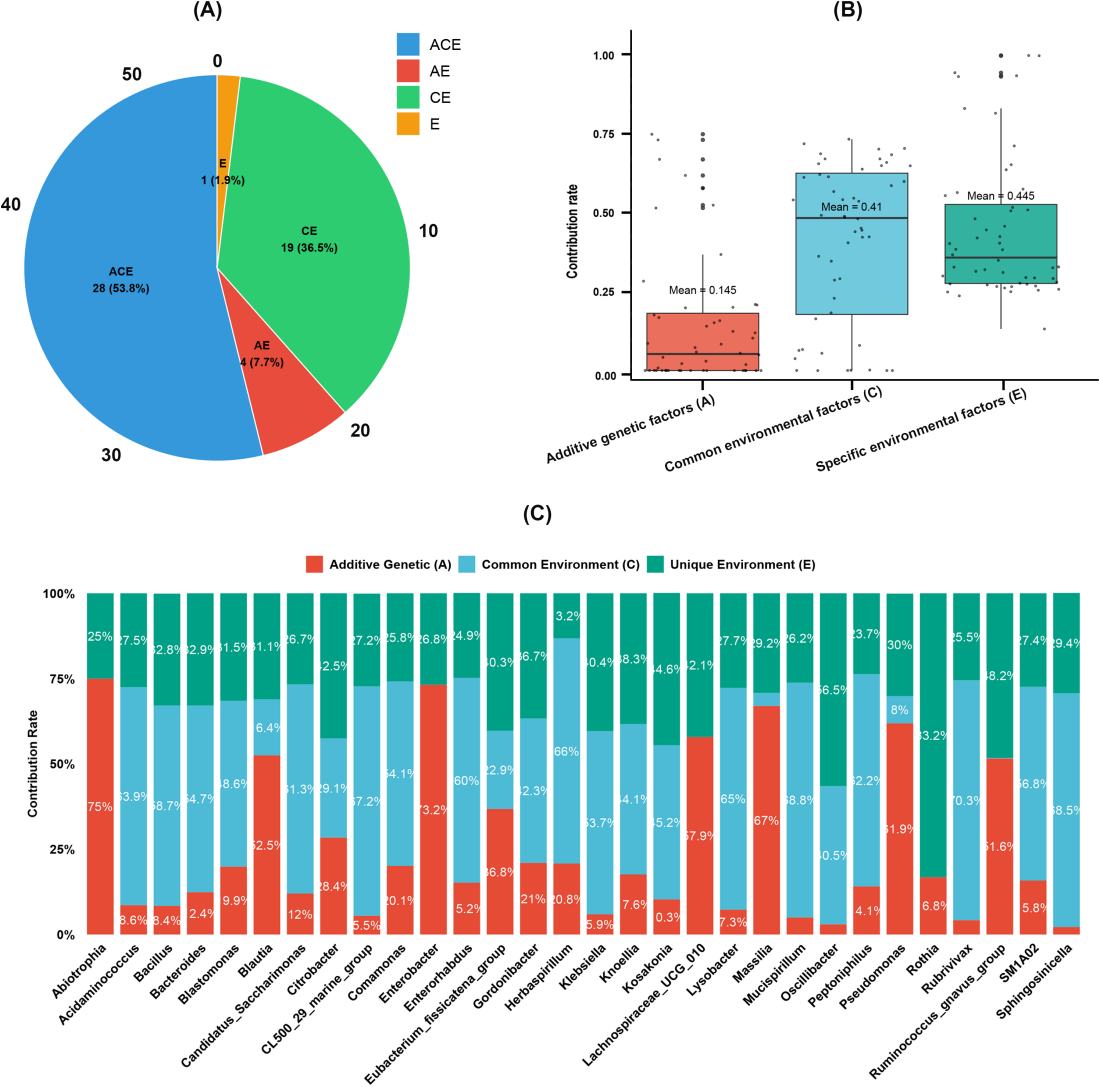
Contribution of genetic and environmental factors on twins’ gut microbiota at 12 months old. **(A)** Distribution of ACE model types for heritable gut microbiota genera. Pie chart showing the classification of 52 heritable gut microbiota genera into three twin-based variance component models: ACE (additive genetic + common environmental + unique environmental effects), CE (common environmental + unique environmental effects), and AE (additive genetic + unique environmental effects). Numbers and percentages in parentheses indicate the count and proportion of genera assigned to each model type. **(B)** Average contribution of variance components to heritable gut microbiota genera. **(C)** Variance component contributions of top 30 heritable gut microbiota genera.

ACE models were used to determine how the environment and genetic factors impact microbiota genera, with 119 families having both twins’ data on gut microbiota included, together controlling for group and twin sex variables. As a result, among the 188 microbiota genera, only 52 genera had genetic variation (**Supplementary Table S1**). There were four types of ACE model types among the 52 genera, as shown in **Figure 2A**, and the top 30 taxa of genetic effects were displayed in **Figure 2B**, and the averaged contributions of all the genera were illustrated in **Figure 2C**. Unfortunately, the 10 group-specific taxa showed no genetic validation in our results.

### Altered fecal metabolites co-occurred with distinguished gut microbiota in PT

**Figure 3:**
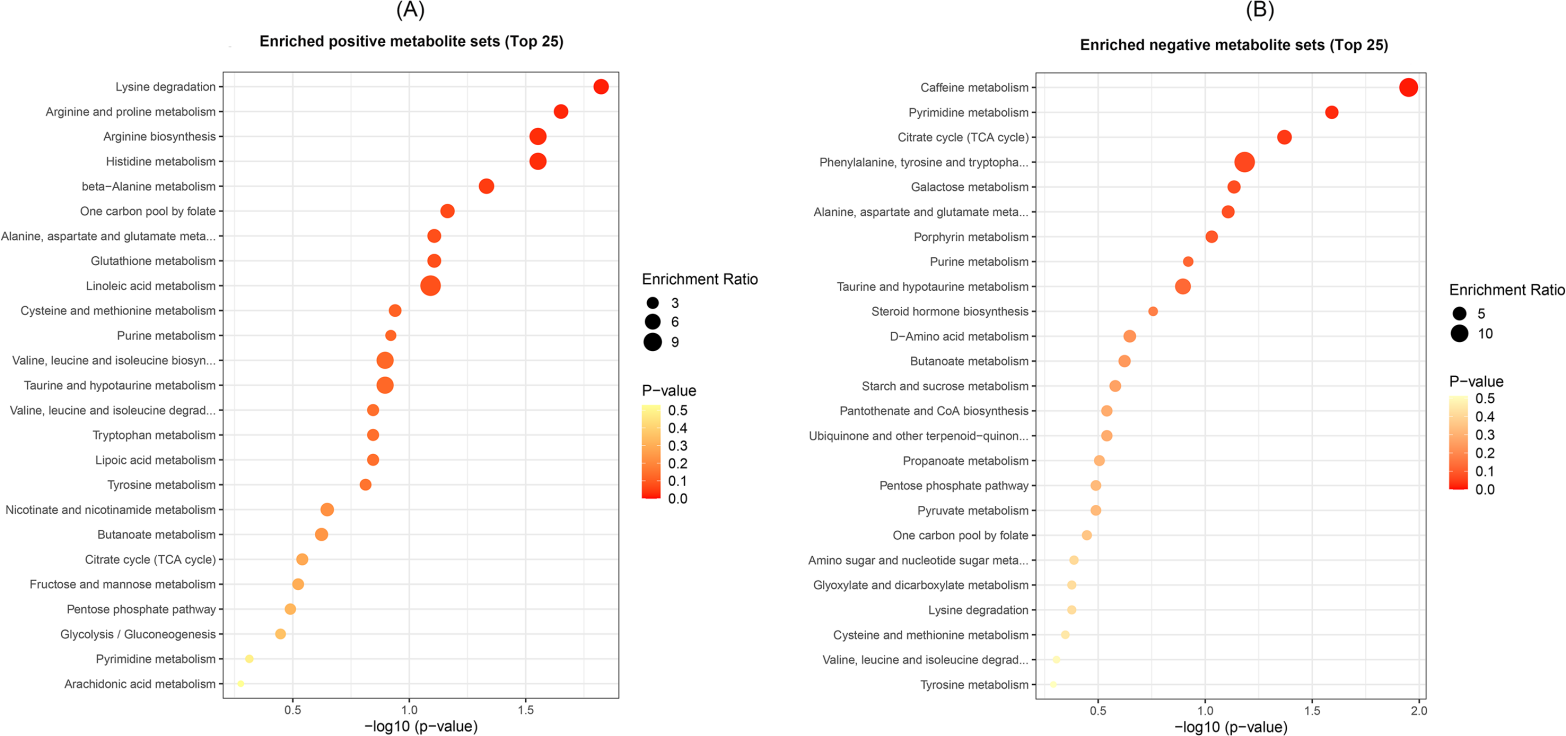
The KEGG enrichment pathways metabolites mainly involved in. Panel **(A)**: the top 25 enriched positive metabolite sets; Panel **(B)**: the top 25 enriched negative metabolite sets. Abscissa variations indicate ratio of the number of differential metabolites in the corresponding pathway to the total number of determined metabolites in that pathway. The bigger the ratio is, the higher enrichment of metabolites are in the pathway. The color of the dots represents the p value of the hypergeometric test. The smaller the value is, the more reliable the test is. The size of the dots represents the number of differential metabolites in the corresponding pathway. The bigger the size of the dots are, the more differential metabolites are enriched in the pathway.

Untargeted metabolomics analysis was simultaneously applied to compare the metabolic signatures among the four groups. Based on GEEs, we compared the metabolic differences among the four groups for all the 2,721 metabolites (1,765 positive and 956 negative metabolites) with twins’ sex, birth weight, delivery mode, and antibiotic use during 12 months of age controlled. The abundance of 394 metabolites significantly differed among the four groups, adjusted *p* < 0.05 (**Supplementary Table S2**). According to the KEGG pathway analyses, these altered metabolites were mainly involved in lysine degradation, arginine and proline metabolism, arginine biosynthesis, histidine metabolism, and beta-alanine metabolism in up-regulated metabolites (*p* < 0.05), and caffeine metabolism, pyrimidine metabolism, and citrate cycle in down-regulated metabolites (*p* < 0.05) (**Figures 3A, B**).

We explored the potential correlations of abundances of the 10 differential taxa and 394 fecal metabolites. Significant correlations between taxa and metabolites with correlation values greater than 0.2 were documented in red in **Supplementary Table S3**.

### Gut microbiota and metabolites correlated with twins’ physical and neurocognitive development in PT

**Figure 4:**
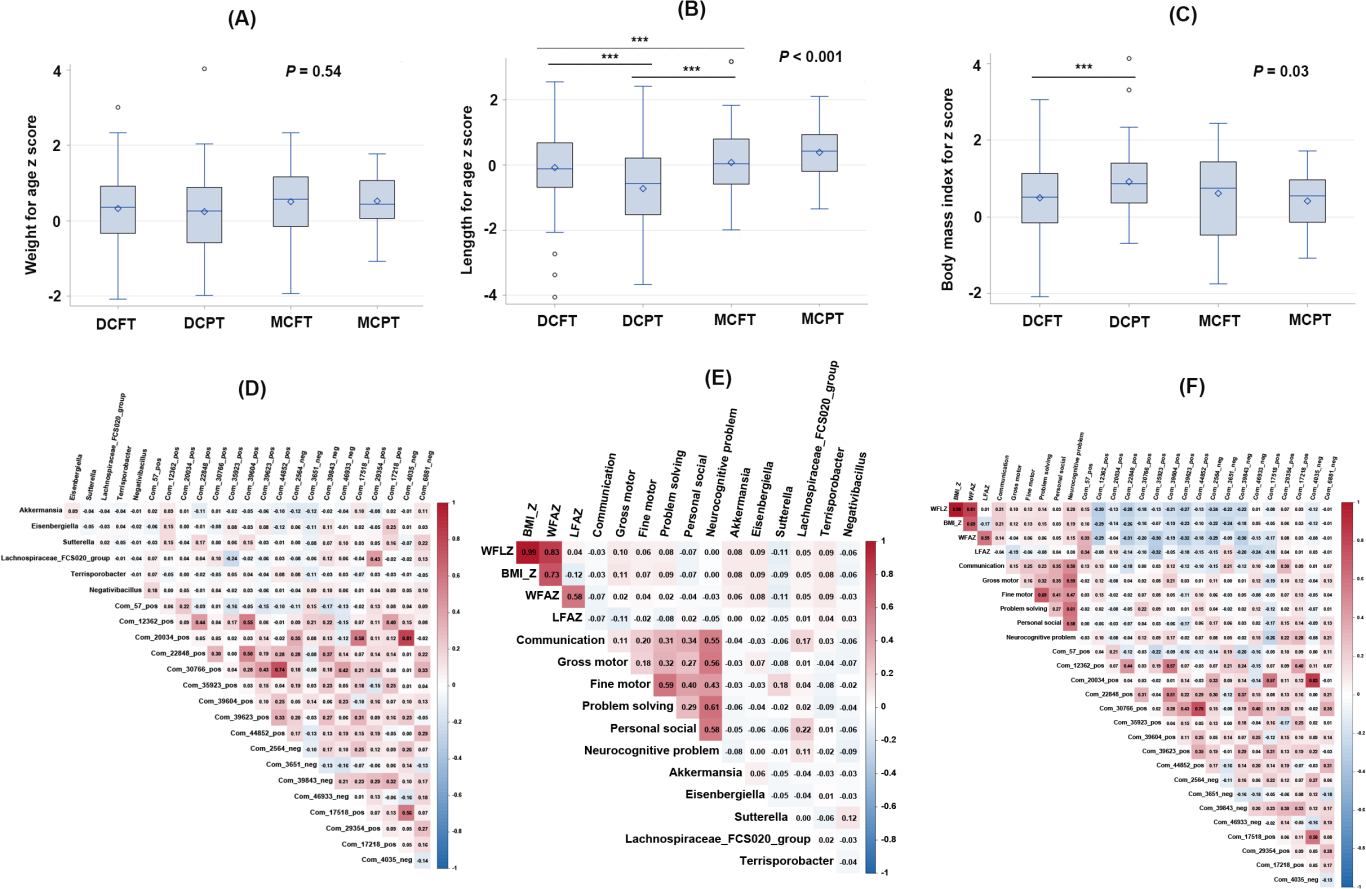
The comparison of physical indicators among the four groups and correlations between microbiota taxa, metabolites, and developmental indicators. DCFT refers to dichorionic-diamniotic full-term twins; DCPT refers to dichorionic-diamniotic preterm term twins; MCFT refers to monochorionic-diamniotic full-term twins; and MCPT refers to monochorionic-diamniotic preterm term twins. LFAZ refers to length for age z-score; WFAZ refers to weight for age z-score; WFLZ refers to weight for length z-score; BMI_Z refers to body mass index for age z-score; ***refers to *p* < 0.001. **(A)** Averaged weight for age z-score among the four twin groups; **(B)** Averaged length for age z score among the four groups; **(C)** Averaged body mass index for z score among the four twin groups; **(D)** Correlation heatmap of group specific gut microbiota genera and fecal metabolites using partial correlation analysis. Confounding factors were twin pair sex, birth weight and delivery mode. The color gradient (blue to red) represents correlation strength (negative to positive), and white cells indicate non-significant correlations; **(E)** Correlation heatmap of gut microbiota genera, physical growth, and neurodevelopmental indicators using partial correlation analysis. Confounding factors were twin pair sex, birth weight and delivery mode. The color gradient (blue to red) represents correlation strength (negative to positive), and white cells indicate non-significant correlations; **(F)** Correlation heatmap of fecal metabolites, physical growth, and neurodevelopmental indicators using partial correlation analysis. Confounding factors were twin pair sex, birth weight and delivery mode. The color gradient (blue to red) represents correlation strength (negative to positive), and white cells indicate non-significant correlations.

Results from ANOVA found no significant difference was found in WFAZ among the four groups (**Figure 4A**). We found that twins in the DCPT group have the smallest LFAZ compared with the other three groups (**Figure 4B**), and the BMI_Z in the DCPT twins was larger than that in the DCFT twins (**Figure 4C**). Results from *chi*-square tests showed that twins in the MCFT group have the highest risks for communication problems; no significant difference was found in the other four domains among the four groups (**Table 3**). In association analyses, among the 10 microbiota taxa, only two taxa, *Akkermansia* and *Eisenbergiella*, were associated with WFAZ or BMI_Z (adjusted *p* < 0.05); six specific gut microbiota genera, *Akkermansia*, *Eisenbergiella*, *Sutterella*, *Lachnospiraceae FCS020 group*, *Terrisporobacter*, and *Negativibacillus*, were associated with neurocognitive development variables (adjusted *p* < 0.05, **Table 4**). Thus, the 6 microbiota genera, shaped by the gestational age and chorionicity, were set as the candidate microbiota that might influence twins’ physical and neurocognitive development at 12 months of age. Similarly, 18 candidate metabolites were filtered out from the 394 metabolites significantly different between the four groups which were associated with physical and neurocognitive development variables. Correlations between the candidate taxa and metabolites and between them and physical and neurocognitive development are shown in **Figures 4D–F**. Finally, based on the KEGG pathway database (https://www.genome.jp/kegg/compound/), among the 18 metabolites, the functional pathways were found in only three metabolites; Morphine (Com_22848_pos, KEGG ID: C01516), Nicotinuric acid (Com_29354_pos, KEGG ID: C05380), and Catechin (Com_2564_neg, KEGG ID: C06562). Among them, morphine functioned in the isoquinoline alkaloid biosynthesis (map00950), drug metabolism—cytochrome P450 (map00982), biosynthesis of alkaloids derived from the shikimate pathway (map01063), metabolic pathways (map01100), biosynthesis of secondary metabolites (map01110), and neuroactive ligand-receptor interaction (map04080); nicotinuric acid functioned in nicotinate and nicotinamide metabolism (map00760); and catechin functioned in flavonoid biosynthesis (map00941), biosynthesis of phenylpropanoids (map01061), and biosynthesis of secondary metabolites (map01110).

**Table 3:** Neurocognitive development for twins with different gestational age and chorionicity (number of ND/DD).

Variable	Total sample size (*n* = 238)					Participants with metabolism data (*n* = 134)				
	DCFT group	DCPT group	MCFT group	MCPT group	*P*-value	DCFT group	DCPT group	MCFT group	MCPT group	*P*-value
Communication	103/11	40/6	27/7	16/0	0.08	62/8	24/2	12/4	8/0	0.20
Gross motor	105/9	39/7	27/7	15/1	0.17	62/8	23/3	10/6	7/1	0.12
Fine motor	107/7	41/5	31/3	16/0	0.32	63/7	23/3	14/2	8/0	0.60
Problem solving	102/12	36/10	29/5	16/0	0.04	59/11	22/4	12/4	8/0	0.30
Personal social	101/13	42/4	26/8	15/1	0.20	60/10	24/2	11/5	7/1	0.26
Neurocognitive problem	84/30	29/17	20/14	15/1	0.02	46/24	18/8	8/8	7/1	0.28

ND/DD refers to normal development/development delay; DCFT refers to dichorionic-diamniotic full-term twins; DCPT refers to dichorionic-diamniotic preterm term twins; MCFT refers to monochorionic-diamniotic full-term twins; and MCPT refers to monochorionic-diamniotic preterm term twins; preterm term was defined as delivery with a gestational age less than 37 weeks; false discovery rate was used for *p-*values.

**Table 4:** Associations between the gestational age and chorionicity specific gut microbiota genera and physical and neurocognitive development.

Variable	WFAZ	LFAZ	BMI_Z	Communication	Gross motor	Fine motor	Problem solving	Personal social	Neurocognitive problem
Akkermansia	1.76^*^	0.20	2.34^*^	−0.44^*^	−0.39	−0.18	−0.44	−0.59^*^	−1.15^*^
Clostridium_sensu_stricto_1	−0.17	−2.34	1.80	−1.04^*^	−0.61	−0.73	0.62	−1.00	−0.91
Collinsella	4.65	2.18	4.30	−0.08	1.09	1.67	0.92	3.32	1.08
Eisenbergiella	16.88	4.49	20.04^*^	−2.21	6.57	−2.76^*^	−4.37^*^	−2.86^*^	1.17
Enterorhabdus	−896.08	114.28	−1342.18	−155.54	−374.75	49.62	64.89	212.31	34.76
Lachnospiraceae_FCS020_group	414.38	120.89	430.84	327.83^*^	−2.76	92.88	95.55	435.00^*^	336.59^*^
Negativibacillus	−438.42	−218.98	−441.28	−203.80	−295.21^*^	−41.79	−168.06	-265.41^*^	−512.06^*^
Solobacterium	−405.54	183.17	−682.19	503.20	275.40	245.37	229.42	228.08	1418.45
Sutterella	−46.13	−20.80	−47.46	−4.74	−12.91^*^	24.42	−4.40	−9.73^*^	1.86
Terrisporobacter	195.22	66.78	229.22	43.08	−33.27	−68.96^*^	−90.78^*^	36.66	−12.11

WFAZ, weight for age z-score; LFAZ, length for age z-score; and BMI_Z, body mass index z-score; GEE models were used with twins’ sex, birthweight, delivery mode and antibiotic used within the first 12 months of life adjusted, with twin family as random effects; *refers to bonferoni corrected *p-*value less than 0.05.

## Discussion

To our knowledge, this is the first study exploring the relationship between gestational age and chorionicity and 12-month-old gut microbiota, metabolic, and physical, and neurobehavioral development in twins. We found a significant difference in the relative abundance of 10 microbiota genera with no genetic effects found and in the abundance of 394 metabolites among the gestational age and chorionicity specified four groups in 12-month-old twins. In addition*, Akkermansia* and *Eisenbergiella* were associated with WFAZ or BMI_Z; apart from them, *together with Sutterella*, *Lachnospiraceae FCS020 group*, *Terrisporobacter*, and *Negativibacillus, they* were associated with infants’ neurocognitive development. Among the 394 specified metabolites, most are enriched in amino acid metabolism pathways (including lysine degradation, arginine and proline metabolism, etc.) and the citrate cycle. And 18 metabolites were filtered to be significantly associated with infants’ physical and neurocognitive development.

Gestational age at birth and chorionicity are two main signature features of twins. Their combination may lead to different maternal and neonatal birth outcomes and influence infants’ bacterial colonization (D’Antonio et al., 2017). For instance, studies on PT reported that microbiota in PT displayed delayed maturity with prolonged membership of facultative anaerobic bacteria compared to that of the predominantly strict anaerobic community of term infants (Rao et al., 2021; Healy et al., 2022). For another, the difference in chorionicity may lead to the difference in fetus gut microbiota establishment, thus influencing microbiota assembly after birth (Yang et al., 2022). Our study found significantly higher richness of relative abundance in the MCPT group than in the MCFT and DCFT groups. In addition, higher Bray–Curtis distances were found in PT and dichorionic-diamniotic twins. Results from PCoAs also displayed similar results. All these results reflected that PT might influence gut microbiota structure at 12 months of age after controlling for the effect of genetic influence (adjusting for chorionicity) (Fouhy et al., 2019; Sugino et al., 2021). We also filtered 10 specific gut microbiota genera for the four groups by comparison analyses. Among these genera, the cumulative frequency in the relative abundance of the genera *Clostridium sensu stricto1, Collinsella*, and *Akkermansia* was significantly higher in the dichorionic-diamniotic twin groups than that in the monochorionic-diamniotic twin groups. These results indicated that chorionicity plays a significant role in shaping the gut microbiota in 12-month-old twins, consistent with the results of Yang et al. for twin neonates who suffered from fetal growth restrictions (Yang et al., 2022). To further confirm the effects of genes and environment on gut microbiota, we assessed the influence of genetic and environmental factors on all the effective microbiota genera. Genetic effects were detected in only 52 taxa, with all 10 distinct microbiota having no genetic effects. These results of ACE models reinforced the perspective that compared to genetics, environmental factors, including PT and chorionicity, may have greater impacts on gut microbiota at 12 months of age (Bäckhed et al., 2015; Rautava, 2022). It is known that preterm neonates experience a number of unique challenges to the establishment of their microbiota (Hill et al., 2017). Unique events in the hospital, like more likely to be Caesarean section, more time in the hospital after birth, more likely to receive formula milk, may influence the pace of microbial acquisition and more likely of antibiotic exposure, shorter breastfeeding duration, and later supplementary food et al. after leaving the hospital environment, which may influence the sequence of microbial acquisition, are the main determinants of the bacterial community profile in preterm infants compared with full-term births (La Rosa et al., 2014). Chorionicity categorizes distinct intrauterine environmental states, where the profiles of adverse intrauterine conditions differ according to chorionicity subtypes. Since birth weight is closely linked to these adverse environments and also serves as an essential factor shaping the infant microbial balance, chorionicity is likely to influence the infant microbiota indirectly via its effect on birth weight.

This study also detected fecal metabolites as a functional readout of gut microbiota. We identified 394 metabolites that significantly differed in abundance among the four groups. With KEGG analyses, the 394 metabolites were categorized into 38 main metabolism pathways. Among the metabolites’ potential functional pathways, significant effects were filtered in eight functional pathways: lysine degradation, arginine and proline metabolism, arginine biosynthesis, histidine metabolism, beta-alanine metabolism, caffeine metabolism, pyrimidine metabolism, and citrate cycle. As it is, significant pathways of most metabolites involved in were amino acid metabolism processes, which was consistent with a study of over 3.3 million newborns on amino acids and acylcarnitines and birth weight and gestational age in China (He et al., 2025) and findings from Nilsson et al. evaluating the association between serum metabolomics and highly premature infants (Nilsson et al., 2022). Our results suggested that gestational age at birth and chorionicity had an impact on infant metabolism, especially on amino acid metabolism and the citrate cycle. More research is needed to understand the mechanisms underlying these correlations.

The topics of PT, gut microbiota and metabolism, and child growth are not uncommon, but studies linking all of these topics together while accounting for genetic effects are lacking. Twins are a unique population in research on environmental and genetic effects. Jing Yang et al. conducted a twins study on this topic. They focused on fetal growth restriction (FGR) twins and discussed the twin neonates’ gut microbiota and metabolism and their effects on physical and neurobehavioral developments. They found that twins with FGR have dysbiotic gut microbiota and metabolic alterations, and the altered fecal cysteine level is positively correlated with the physical and neurocognitive developments of twins at 2 or 3 years of age (Yang et al., 2022). We used this study design to investigate the relationship between PT and chorionicity, distinct gut microbiota and metabolites, and twins’ physical and neurobehavioral growth at 12 months of age. A lower abundance of *Akkermansia muciniphila* is associated with obesity in both mouse models and in humans (Cani et al., 2022; Zhang et al., 2025). A commonly recognized mechanism is that *Akkermansia muciniphila* could contribute to the maintenance of a healthy gut barrier, then regulating immunity and limit the onset of inflammation, thereby preventing obesity (Liu et al., 2025; Zhang et al., 2025). In our study, we found positive associations between *Akkermansia* and physical growth indicators WFAZ and BMI_Z, which is inconsistent with previous findings. In addition, *Eisenbergiella* was also positively correlated with physical growth indicators. A study on two cohorts found *Eisenbergiella* is negatively associated with a healthy lifestyle (Dong et al., 2025), and a multi-omics integration reveals that *Eisenbergiella* has the potential to be an independent biomarker for atherosclerosis except for disease like hypertension, type 2 diabetes, and obesity (Shi et al., 2025). The inconsistent results may be attributed to the population specificity of this study: previous research focused on adults with mature gut microbiota, whereas this study enrolled infants whose gut microbiota is still developing, potentially leading to differences in microbial physiological functions. Evidence from population-based studies and mechanistic studies in animals has shown that the commensal microbiome plays a crucial role in neurodevelopment during early life (Cox et al., 2014; Carlson et al., 2018; Wu et al., 2025). In our study, we found six microbiota taxa that were associated with neurocognitive development; most of them were not reported previously. Therefore, studies are needed to confirm our findings. Among the 18 metabolites that are associated with physical growth and neurocognitive development, three metabolites participated in functional pathway.

This is the first study to systematically explore the combined effects of gestational age and twin chorionicity on gut microbiota, fecal metabolome, and infants’ physical/neurobehavioral development at 12 months of age. It fills the gap in existing literature that lacks integrated analysis of these interconnected topics while accounting for genetic confounding factors. The twin study design enables precise comparison of the independent and interactive effects of PT and intrauterine environment on the outcomes of interest. In addition, heritability estimation using the ACE model allowed us to confirm the genetic effects on the gut microbiota, with complementary analyses performed based on zygosity and chorionicity. Moreover, most of the gut microbiota and metabolites that were significantly differentiated across groups stratified by gestational age and chorionicity and associated with physical growth and neurodevelopmental indicators were identified for the first time in this study, warranting further research to validate these findings. Apart from the advantages we have, there are several limitations in our research. First, as it showed in our study, genetic effects functioned on infants’ gut microbiota. However, we did not evaluate the exact gene or locus that may influence gut microbiota. Further studies are needed in this area. Second, the number of infants in MCFT and MCPT groups are relatively small, and there is a reduction in sample size when analyzing metabolic data, from 119 pairs to 67 pairs, which may reduce the power to identify some more gut microbiota and metabolites that are correlated with environmental effects. Third, almost all twins were delivered by Cesarean section, which may lack generalizability to twins born vaginally. Fourth, we did not collect data about antibiotics received at birth, which may influence the initial gut microbiota. Last, PT in our study was averaged as 35.33 gestational weeks; very or extremely PT (gestational age less than 32 or 28 weeks) is a lack, which may have a great impact on gut microbiota and infant growth.

## Data Availability

The datasets presented in this study can be found in online repositories. The names of the repository/repositories and accession number(s) can be found below: https://ngdc.cncb.ac.cn/gsa, CRA007379.
