# Supplementary figures and images for: Preterm birth alters the gut microbiota, metabolome and health outcomes of twins at 12 months of age
Source: Front Cell Infect Microbiol. 2026 Jan 21;15:1700965. doi: 10.3389/fcimb.2025.1700965 (PMC12868185; doi:10.3389/fcimb.2025.1700965)

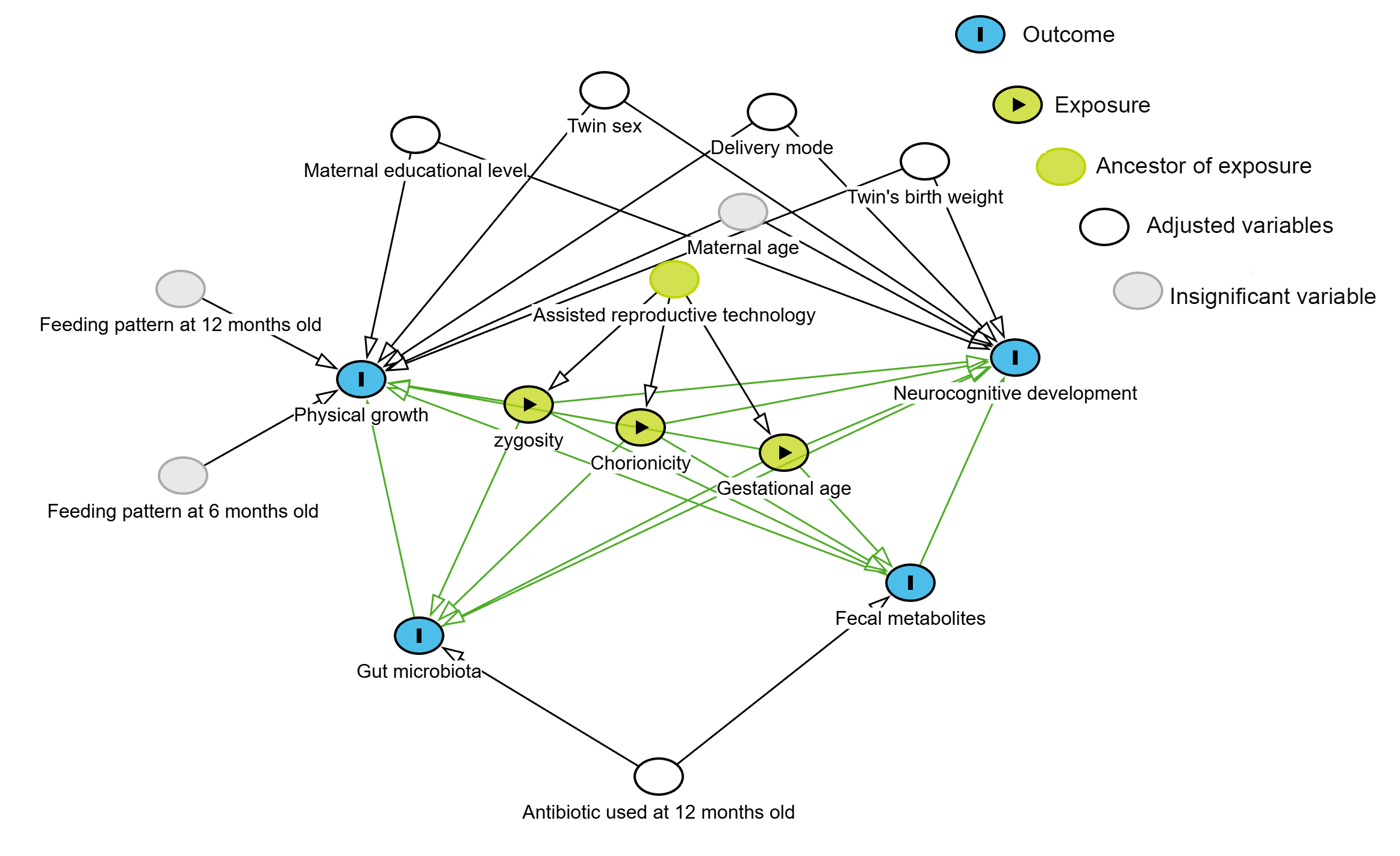

Supplement: Supplementary file 1 [file Image1.png]

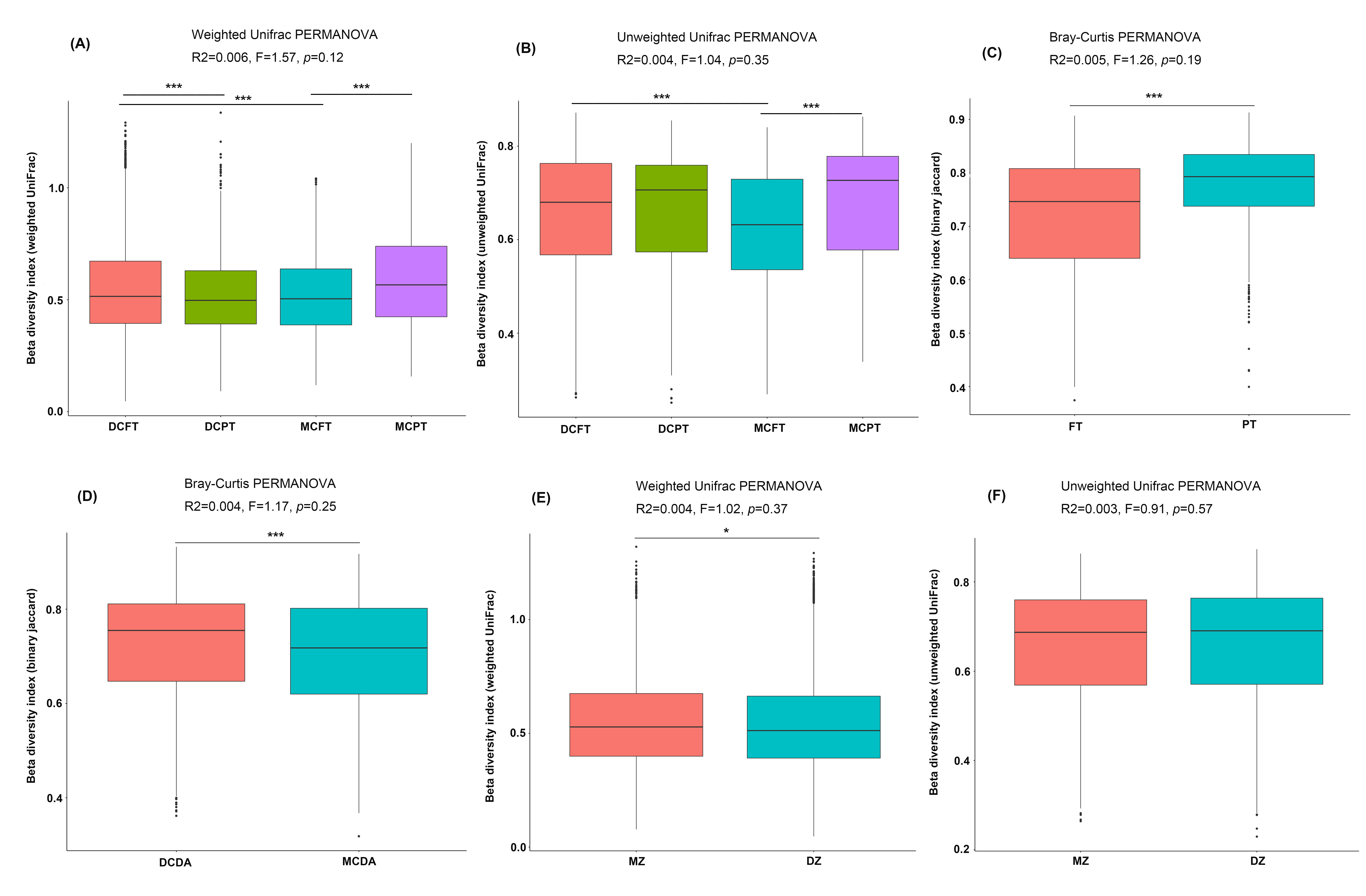

Supplement: Supplementary file 2 [file Image2.tif]

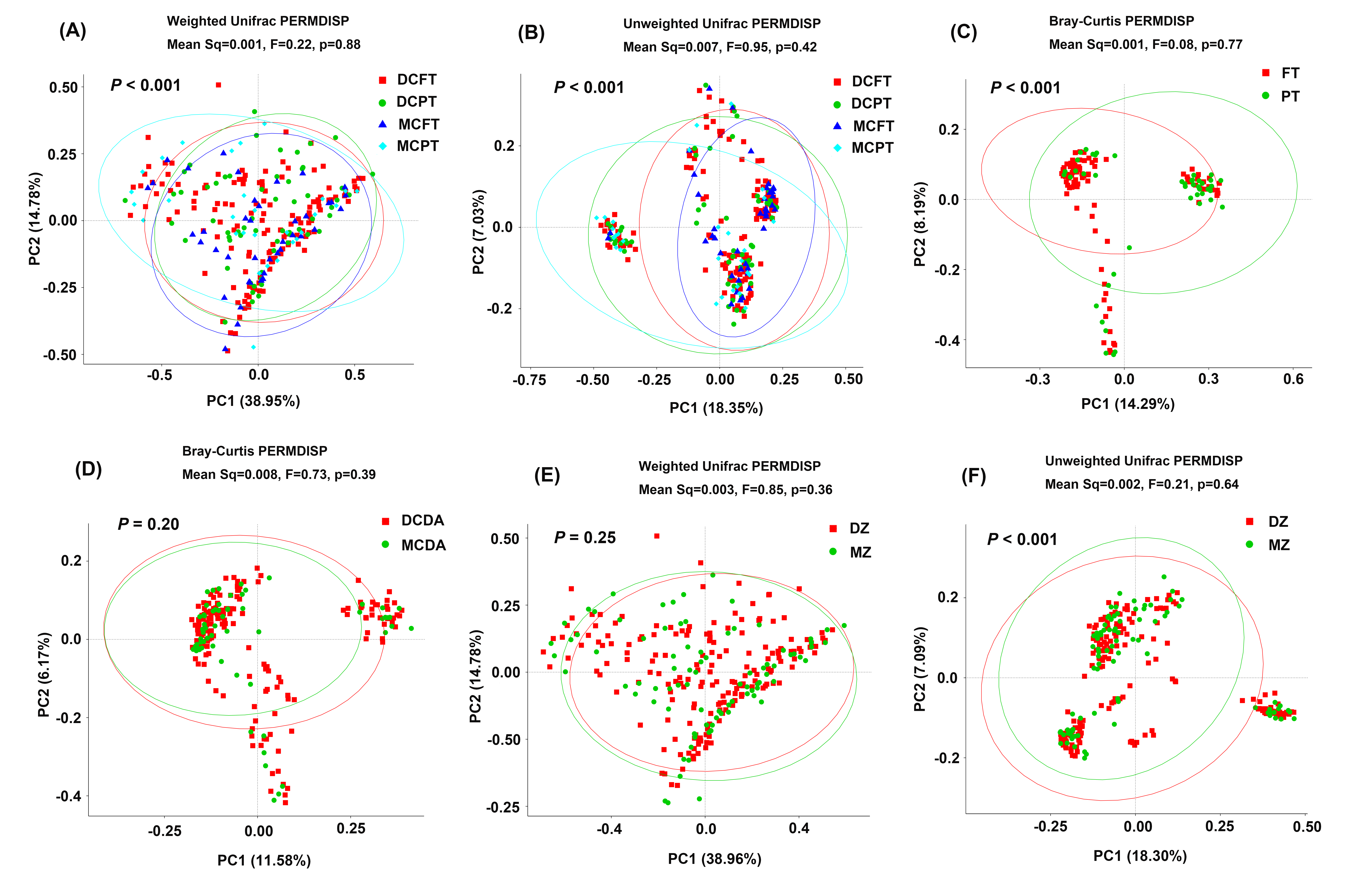

Supplement: Supplementary file 3 [file Image3.tif]

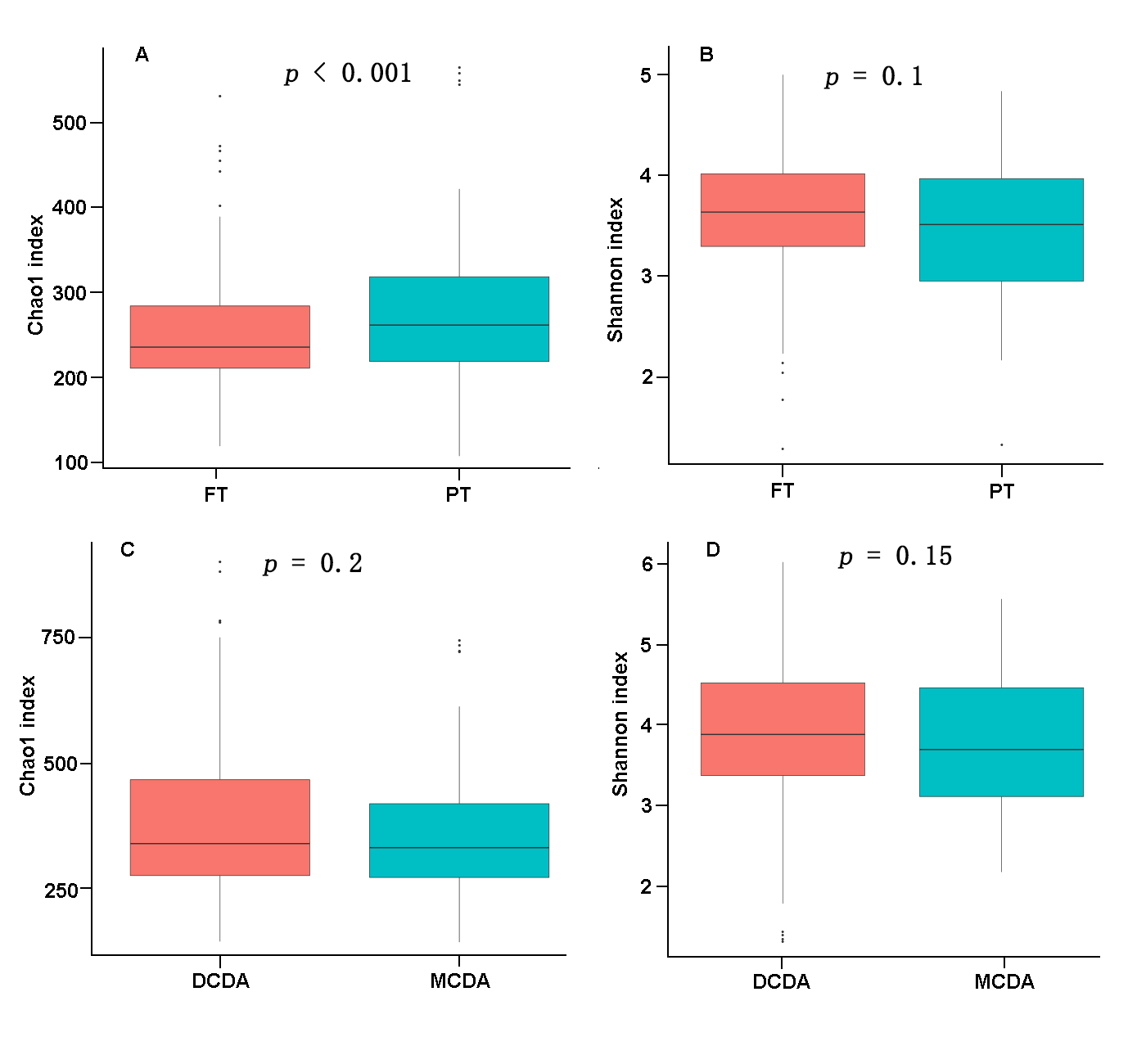

Supplement: Supplementary file 4 [file Image4.tif]
