# Supplementary material for: Preterm birth alters the gut microbiota, metabolome and health outcomes of twins at 12 months of age
Source: Front Cell Infect Microbiol. 2026 Jan 21;15:1700965. doi: 10.3389/fcimb.2025.1700965 (PMC12868185; doi:10.3389/fcimb.2025.1700965)
Supplement: Supplementary file 5 [file Supplementaryfile1.doc]

**Supplementary materials**

**Supplementary figure legends**

**Supplemental Figure 1. The directed acyclic graph of covariates and BPs and infant growth.**

**Supplemental Figure 2. The alpha diversity analyses of the gestational age subgroup and chorionicity subgroup.**

Note: FT refers to full-term birth group; PT refers to preterm birth group; DCDA refers to dichorionic-diamniotic birth twins; MCDA refers to monochorionic-diamniotic birth twins.

It showed that the Chao1 index was significantly higher in the preterm birth group than in the full-term birth group. However, no significantly difference was found for Chao1 index in the chorionicity subgroup. The differences of Shannon index were not significant for both gestational age subgroup and chorionicity subgroups as shown in Panel B and D.

**Supplemental Figure 3. Beta diversity of gut microbiota across twin subgroups stratified by chorionicity, gestational age, and zygosity.**

Note: DCFT refers to dichorionic-diamniotic full-term twins; DCPT refers to dichorionic-diamniotic preterm term twins; MCFT refers to monochorionic-diamniotic full-term twins; and MCPT refers to monochorionic-diamniotic preterm term twins; FT refers to full-term birth group; PT refers to preterm birth group; DCDA refers to dichorionic-diamniotic birth twins; MCDA refers to monochorionic-diamniotic birth twins; MZ refers to mono-zygotic twins; DZ refers dizygotic twins.

Panels A-C: Beta diversty (weighted/unweighted Unifrac, Bray-Curtis) of gut microbiota among subgroups defined by chorionicity and gestational age; *** indicates statistical significance at p < 0.001; PERMANOVA results (R^2^: proportion of variance explained by grouping; *F*, *p*: test statistics) are shown above each panel.

Panel D: Bray-Curtis beta diversity comparison between DCDA and MCDA subgroups; *** indicates statistical significance at p < 0.001; PERMANOVA metrics are presented above the panel.

Panel E-F: Weighted/unweighted UniFrac beta diversity of gut microbiota between MZ and DZ twin subgroups; *** indicates statistical significance at p < 0.001; PERMANOVA metrics are presented above the panel.

**Supplemental Figure 4. Principal Coordinate Analysis ordinations with PERMDISP test across twin subgroups.**

Note: DCFT refers to dichorionic-diamniotic full-term twins; DCPT refers to dichorionic-diamniotic preterm term twins; MCFT refers to monochorionic-diamniotic full-term twins; and MCPT refers to monochorionic-diamniotic preterm term twins; FT refers to full-term birth group; PT refers to preterm birth group; DCDA refers to dichorionic-diamniotic birth twins; MCDA refers to monochorionic-diamniotic birth twins; MZ refers to mono-zygotic twins; DZ refers dizygotic twins.

**Supplementary Methods**

**Untargeted metabolomics analysis of fecal samples**

Fecal samples of 12 months old twins were individually grounded with liquid nitrogen. The well-vortex suspended the homogenate with prechilled 80% methanol and 0.1% formic acid. The samples were incubated on ice for 5 min and centrifuged at 15000 rpm, 4°C for 20 minutes. Next, the supernatant was diluted to a final concentration containing 53% methanol by LC-MS grade water. The samples were transferred to a new Eppendorf tube and centrifuged for 10 minutes at 15000 rpm, 4°C. Finally, the supernatant was injected into the LC-MS/MS system analysis.

UHPLC-MS/MS analyses were performed using a Vanquish UHPLC system (Thermo Fisher, Germany) coupled with an Orbitrap Q Exactive^TM^ HF-X mass spectrometer (Thermo Fisher, Germany) in Novogene Co., Ltd. (Beijing, China). Samples were injected onto a Hyperil Gold column (100 × 2.1 mm, 1.9 μm) using a 17-min linear gradient at a 0.2 mL/min flow rate. The eluents for the positive polarity mode were eluent A (0.1% FA in Water) and eluent B (Methanol). The eluents for the negative polarity mode were eluent A (5 mM ammonium acetate, pH 9.0) and eluent B (Methanol). The solvent gradient was set as follows: 2% B, 1.5 min; 2-100 % B, 12.0 min; 100 % B, 14.0 min; 100-2 % B, 14.1 min; 2 % B, 17 min. The Q Exactive^TM^ HF-X mass spectrometer was operated in positive/negative polarity mode with a spray voltage of 3.2 kV, a capillary temperature of 320 °C, a sheath gas flow rate of 40, and an aux gas flow rate of 10 arb.

The raw data files generated by UHPLC-MS/MS were processed using Compound Discoverer 3.1 (CD 3.1, Thermo Fisher, Germany) to perform peak alignment, peak picking, and quantitation for each metabolite. First, the main parameters were set as follows: retention time tolerance, 0.2 minutes; actual mass tolerance, 5 ppm; signal intensity tolerance, 30%; signal/noise ratio, 3; and minimum intensity, 100000. Then, peak intensities were normalized to the total spectral intensity. Next, the normalized data predicted the molecular formula based on additive ions, molecular ion peaks, and fragment ions. Then, peaks were matched with the mzCloud (<https://www.mzcloud.org/>), mzVault, and MassList databases to obtain accurate qualitative and relative quantitative results. Finally, the metabolites were annotated using the KEGG database (<https://www.genome.jp/kegg/pathway.html>), HMDB database (https://hmdb.ca/metabolites.html), and LIPIDMaps database (<https://lipidmaps.org/>).
